# Cystic lesions in osteoarthritis and osteonecrosis of the femoral head: a comparative analysis of 3D distribution, microstructure, and histology

**DOI:** 10.1302/2046-3758.1410.BJR-2024-0478.R2

**Published:** 2025-10-01

**Authors:** Yiwei Chen, Jiapeng Li, Jiali Lin, Yu Miao, Junhui Yin, Guangyi Li, Changqing Zhang

**Affiliations:** 1 Department of Orthopaedics, Shanghai Sixth People's Hospital Affiliated to Shanghai Jiao Tong University School of Medicine, Shanghai, China; 2 Institute of Microsurgery on Extremities, Shanghai Sixth People's Hospital Affiliated to Shanghai Jiao Tong University School of Medicine, Shanghai, China; 3 Shanghai Medical College, Fudan University, Shanghai, China

**Keywords:** Cystic lesions, Osteoarthritis, Osteonecrosis of the femoral head, Bone remodeling, Osteoarthritis (OA), osteonecrosis of the femoral head (ONFH), femoral heads, bone remodelling, micro-CT scanning, trabecular bone, bone resorption, bone formation, lesions

## Abstract

**Aims:**

Cystic lesions are strongly associated with the pathogenesis and selection of treatment strategies in osteoarthritis (OA) and osteonecrosis of the femoral head (ONFH). However, the differences in cystic lesions arising from these two diseases are not fully understood. This study aimed to delineate the variations in cyst characteristics in the femoral heads of patients with OA and ONFH.

**Methods:**

A total of 45 patients with OA and 105 patients with ONFH who underwent total hip arthroplasty (THA) between September 2014 and December 2021 were recruited in the study. The 3D distribution, microstructure, and histological characteristics of cystic lesions were comprehensively analyzed. Comparative assessments of the microstructural, histomorphometric, and histopathological properties of cystic lesions between OA and ONFH were performed, using micro-CT, decalcified and undecalcified bone histomorphology, and scanning electron microscopy (SEM).

**Results:**

In comparison to ONFH, cystic lesions in OA exhibited a smaller volume and a denser distribution. Despite a common prevalence in the anterior hemisphere in both conditions, ONFH cysts were predominantly located laterally, whereas OA cysts were found mainly medially. In OA, the trabecular bone surrounding the cystic lesions exhibited a more sclerotic microarchitecture, with an increase in bone formation and a decrease in bone resorption at the remodelling level. Histologically, cystic lesions in ONFH demonstrated a higher degree of angiogenesis compared to those in OA.

**Conclusion:**

While cystic lesions in both OA and ONFH were predominantly located in the anterior hemisphere, they exhibited different distribution and involvement characteristics, microstructure and bone remodelling properties, as well as histopathological features.

Cite this article: *Bone Joint Res* 2025;14(10):820–831.

## Article focus

- This study aimed to delineate the variations in cyst characteristics in the femoral heads of patients with osteoarthritis (OA) and osteonecrosis of the femoral head (ONFH), in terms of the microarchitectural, histomorphological, and histopathological aspects.

## Key messages

- Our results indicate that while cystic lesions in both OA and ONFH were predominantly located in the anterior hemisphere, they exhibited different distribution and involvement characteristics, microstructure and bone remodelling properties, as well as histopathological features.

## Strengths and limitations

- We employed clinical imaging, micro-CT, decalcified and undecalcified bone histology, and scanning electron microscopy to elucidate the difference in cyst characteristics in the femoral heads of patients with OA and ONFH.
- The study could enhance the understanding of microstructural, pathological, and histomorphological alterations of cystic lesions in OA and ONFH, thereby fostering a deeper comprehension of the disease and potentially influencing clinical decision-making.
- Dynamic bone remodelling characteristics were not evaluated in the femoral head specimens in this cross-sectional study.
- In vivo micro-CT scanning of the hip joint is currently not feasible. Advancements in CT and MRI with enhanced spatial resolution in the future may offer a solution.

## Introduction

Osteoarthritis (OA) and osteonecrosis of the femoral head (ONFH) are common conditions that lead to substantial morbidity and affect the quality of life for millions of people worldwide. These diseases are characterized by distinct pathologies that result in the breakdown of joint structures and subsequent pain and disability.^1-3^ A key feature in the pathogenesis of both conditions is the development of cystic lesions,^4,5^ which are associated with disease progression and influence therapeutic decision-making.^6,7^

Two potential mechanisms have been proposed for cyst formation. The ‘synovial fluid intrusion theory’ states that cartilage injuries disrupt the calcified tissue barrier between the cartilage and subchondral bone, which leads to synovial fluid intruding into the bone marrow region and forming a cyst.^8,9^ Conversely, the ‘bone contusion theory’ hypothesizes that trabecular bone is primarily damaged by severe mechanical stress, leading to bone absorption and ultimately cyst formation.^10^

In OA, cystic lesions are believed to form due to the disruption of the subchondral bone plate, allowing synovial fluid to infiltrate the bone marrow and cause cyst formation.^11-13^ These cysts can expand, leading to the weakening of the subchondral bone and an increased risk of joint collapse.^14^ In contrast, ONFH is characterized by the death of bone cells in the femoral head, often due to a compromise in blood supply.^15^ The ensuing repair process can lead to the formation of cystic lesions as part of the bone’s attempt to heal the necrotic area.^6^ These lesions are often larger and more destructive than those seen in OA, and are closely associated with the risk of femoral head collapse.^6,16^

Cystic lesions exhibit distinct roles in the evolution of OA and ONFH. Understanding these differences can deepen our knowledge of disease mechanisms and improve treatment strategies. Firstly, recognizing these differences aids in accurate diagnosis and classification, guiding appropriate treatments. For instance, joint-preserving therapies are more likely to be successful in the early stages of ONFH before cyst formation and collapse have occurred. ^17^ Secondly, the presence, size, and location of cystic lesions can provide valuable prognostic information, helping to predict the risk of disease progression and the potential need for joint arthroplasty surgery.^18^ Finally, understanding the formation of cysts offers insights into underlying biological mechanisms, potentially revealing new therapeutic targets.^19,20^ Current research mainly addresses cyst formation and distribution in OA,^4,21^ while studies on ONFH remain limited.^22^ In addition, cyst-related bone remodelling status and pathological alterations are inadequately understood. The three-pillar classification method based on the location of necrotic bone is widely accepted in ONFH research, which is of particular clinical significance in collapse prediction.^23^ The femoral head is divided into three pillars according to the classification system, with the medial, central, and lateral pillars comprising 30%, 40%, and 30% of the femoral head, respectively.^17^ This three-pillar classification system provides a suitable reference for investigating the localization of cystic lesions and their association with articular collapse in ONFH.

This study aims to compare the 3D distribution, microstructure, and histopathological features of cystic lesions in OA and ONFH. We hypothesize that cystic lesions in OA and ONFH differ in size, distribution, and bone remodelling activity, which may have considerable implications for diagnosis and treatment. By using advanced imaging techniques, such as micro-CT and scanning electron microscopy (SEM), coupled with detailed histomorphometric analysis, this study aimed to provide a comprehensive comparison of cystic lesions in OA and ONFH. The findings from this study may improve our understanding of these conditions and contribute to more targeted, effective therapies.

## Methods

### Subjects

Between September 2014 and December 2021, a total of 45 patients with OA and 105 patients with ONFH who underwent total hip arthroplasty (THA) were recruited. Patients with OA exhibited moderate or severe radiological evidence of the disease, according to the Kellgren and Lawrence criteria.^24^ Exclusion criteria for patients with OA were as follows: 1) OA secondary to osteonecrosis; 2) treatment affecting bone metabolism, such as antiresorptive drugs, calcitonin, thyroid hormones, or hormonal replacement therapy; 3) a history of hip osteotomy; and 4) any metabolic or bone disorder other than OA that affects bone metabolism, such as severe renal impairment, thyroid disease, or malignancy. Inclusion criteria for subjects with ONFH were as follows: 1) diagnosis of ONFH according to the Guidelines for clinical diagnosis and treatment of osteonecrosis of the femoral head in adults (2019 version);^25^ and 2) Association Research Circulation Osseous (ARCO) stages II and III.^26^ Exclusion criteria for patients with ONFH included: 1) a history of hip injury or surgery; 2) OA or congenital hip disorders; and 3) primary hip cysts.^22^

OA and ONFH groups were well matched in terms of demographic factors and medical conditions (Table I), except for the age and time since disease onset, which was due to distinct pathogenesis characteristics of the two diseases. Each patient provided informed consent, and the study protocol was approved by the Human Research Ethics Committee of Shanghai Sixth People’s Hospital in accordance with the Helsinki Declaration.^27^

**Table I.** Baseline characteristics of the participants.

Characteristic	OA	ONFH	p-value
**Study participants**			
Number of patients	45	105	
Sex (male/female), n	33/12	78/27	0.903*
Mean age, yrs (SD)	63.20 (10.62)	49.64 (13.38)	< 0.001†
Mean BMI, kg/m^2^ (SD)	23.77 (3.19)	24.27 (3.19)	0.330‡
Smoker, n (%)	8 (17.78)	27 (25.71)	0.292*
Diabetes, n (%)	5 (11.11)	11 (10.48)	0.908*
Hypertension, n (%)	7 (15.56)	19 (18.10)	0.724*
**Disease**			
Number of hips	45	120	
Side (left/right)	22/23	67/53	0.425*
Mean time since the onset of the disease, mths (SD)	84.60 (98.77)	27.08 (49.65)	< 0.001‡

* Chi-squared test.

† Independent-samples *t*-test.

‡ Mann-Whitney U test.

OA, osteoarthritis; ONFH, osteonecrosis of the femoral head.

### Imaging and image analysis

CT images of all patients before THA were collected, using SOMATOM Definition Flash (Siemens, Germany). Scan parameters were as follows: tube voltage of 140 kVp, automatic tube current modulation (90 to 130 mA), display field of view (DFOV) of 35.6 cm, and slice thickness of 0.75 mm. Scanning covered the area between the acetabulum and the lesser trochanter of the femur. All hip joints were reconstructed using the B60s kernel, sharp, 3 mm, for coronal views. The 3D structure of the femoral head and cystic lesions were obtained based on the previously described method.^22^ Data of hip CT images were imported into Mimics software (version 21.0; Materialise, Belgium). The femoral head was first reconstructed based on CT values. By manually tracing the outline of the femoral head at each level, the structural details of the femoral head were refined. On CT images, cystic lesions appeared as low-density, translucent areas, without discernable bone structures.

**Fig. 1:**
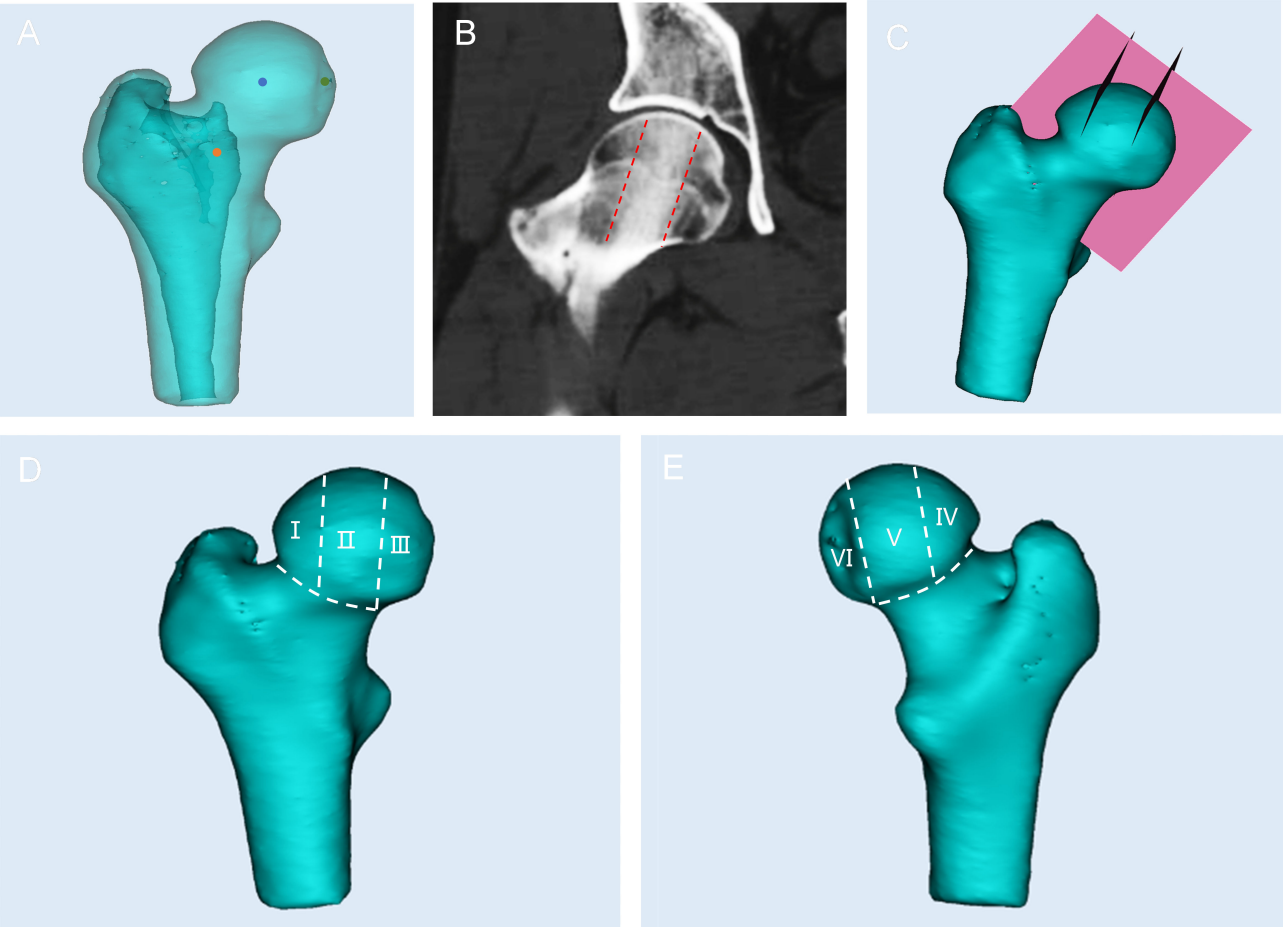
The femoral head and cystic lesions are divided into six regions (I to VI). a) The red point, green point, and blue point indicate the positions of the centre of the calcar, the centre of fovea capitis femoris, and the centre of the femoral head in the 3D structure, respectively. b) In the mediolateral position, the femoral head was divided into three regions by the two red dotted lines parallel to the main trabeculae direction (MTD). c) Plane 1 (pink plane) was obtained, which included the red point, green point, and blue point. Through two dotted lines, two planes perpendicular to Plane 1 are formed, and the two planes are used to divide the femoral head into lateral, central, and medial pillars. d) I to III indicates lateral, central, and medial pillars in the anterior side of femoral head, respectively. e) IV to VI indicates lateral, central, and medial pillars in the posterior side of the femoral head, respectively.

The distribution of cystic lesions was determined by segmenting the femoral head into different regions based on the previous approach.^22^ The process involved the following steps: first, the reference plane was established, encompassing the centres of the femoral head, the fovea capitis femoris, and the femoral neck (Figure 1a). Through two lines parallel to the main trabeculae direction (MTD), two planes perpendicular to the reference plane were formed. The two planes were used to divide the femoral head into lateral, central, and medial pillars, accounting for 30%, 40%, and 30% of the head width, respectively (Figures 1b and 1c). Based on the reference plane, the femoral head was further divided into anterior and posterior sections (Figure 1c). Finally, using these established planes, the femoral head was ultimately segmented into six distinct regions (I to VI) (Figures 1d and 1e).

Based on the six-region segmentation system, the 3D distribution characteristics of cystic lesions were analyzed. The region containing the highest percentage of a specific cystic lesion was defined as the distribution area, given that a cystic lesion may spread across multiple regions simultaneously. Following the region segmentation, the number of cystic lesions in the lateral, central, and medial pillars of the femoral head was determined.

### Micro-CT examination

Femoral head specimens were placed in -80°C freezers after THA surgery. The specimens were subsequently scanned using the high-resolution micro-CT system (CT100; Scanco Medical, Switzerland) using the scan settings listed below: X-ray tube voltage, 70 kV; tube current, 200 μAs; exposure time, 300 ms; projection number, 1,000; and voxel size, 73.6 μm. The length of the scan was approximately 49 mm, yielding 1,200 slices and a 45-minute scan period. In the present investigation, a bone cyst was defined as a space more than 1 mm in diameter, and micro-CT coronal images were analyzed.^28,29^ Cystic lesions in ONFH were characterized as regions related to necrotic lesions, which were different from bone defect areas caused by subchondral fracture or collapse (Supplementary Figure aa).^29^ The femoral head and cystic lesions were modelled in three dimensions (Supplementary Figures ab to ae).

**Fig. 2:**
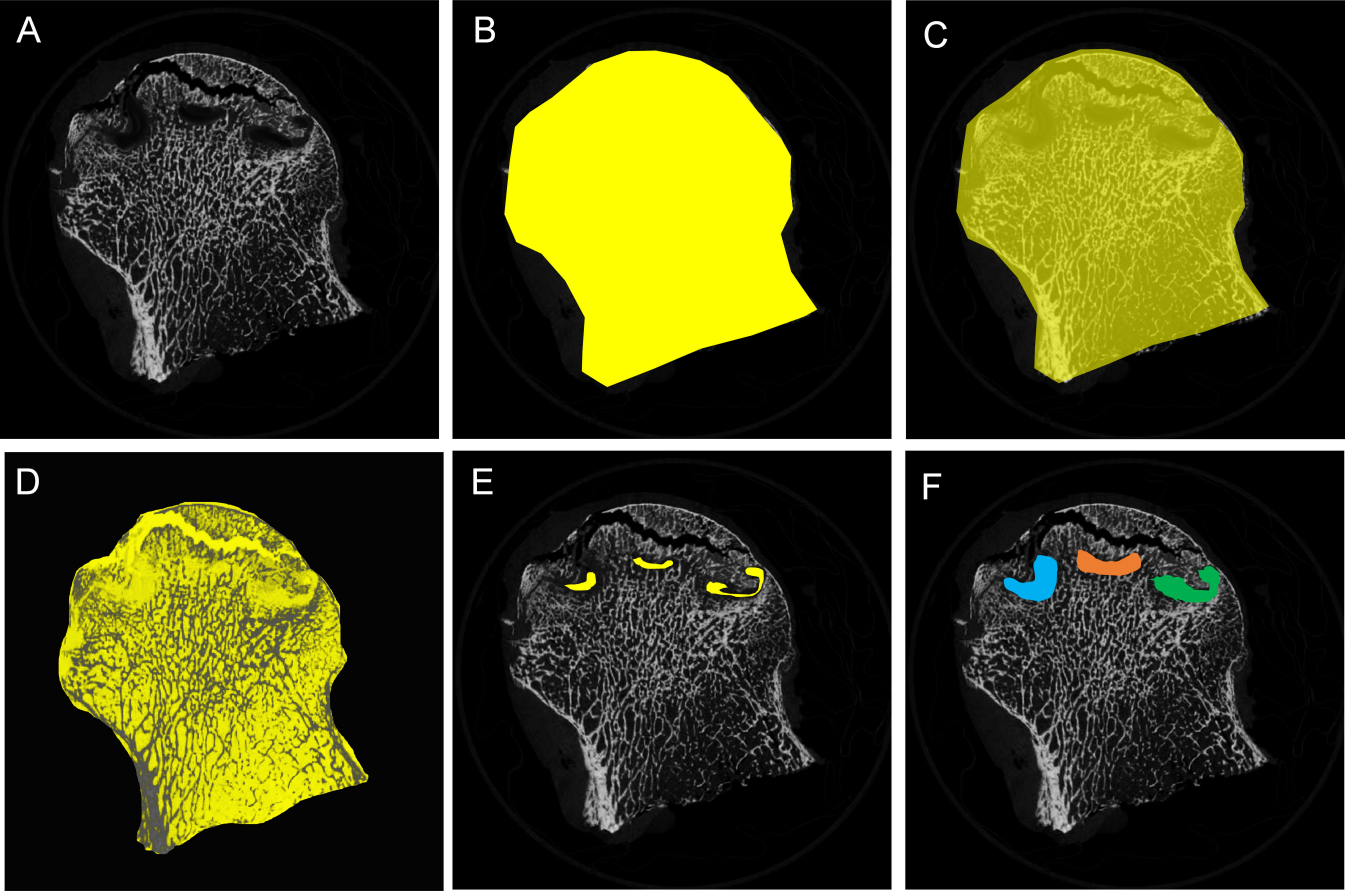
Quantification of cystic lesions defined as areas more than 1 mm in diameter using the built-in software. a) A coronal micro-CT image. b) Bone regions are represented after binarizing CT images using a bone threshold, encompassing the bone trabeculae and the cortical shell. c) The whole of the femoral head is illustrated. d) Empty regions not involving bone are illustrated by subtracting the bone regions from the total region. e) Empty regions less than 1 mm are allowed to erode from the surface three-dimensionally in increments of 0.5 mm in order to identify empty regions larger than 1 mm in diameter. f) The remaining empty areas were dilated on their surfaces by 0.5 mm increments to return them to their original shape in the procedure described above.

On each coronal micro-CT slice, cystic lesions were extracted and bone microstructure was measured using the built-in software in accordance with a previously described approach (Figure 2).^7,29^ In order to distinguish between mineralized tissue and soft-tissue, a consistent global threshold range (90 to 255) was established using grayscale histogram analysis and empirical observations.^30^ Bone regions were visualized by binarizing CT images using the threshold, including the bone trabeculae and the cortical shell (Figure 2b). The entire area was then represented (Figure 2c). By extracting bone tissue out of the overall region, empty spaces were illustrated (Figure 2d). Empty regions of less than 1 mm in diameter were removed and a 3D erosion of the remaining vacant areas was subsequently conducted, incrementally reducing the size by 0.5 mm from the surface (Figure 2e). A 0.5 mm incremental dilation was applied to restore the remaining empty areas to the original shape, as previously outlined (Figure 2f).^7^ The following cyst number and volume-related parameters were measured: total number of all cysts in the subchondral bone region (Cyst.N), total volume of all cysts in the subchondral bone region (Cyst.V) (mm^3^), average volume of a cyst (CystV.Ave) (mm^3^), minimum volume of a cyst (CystV.Min) (mm^3^), and maximum volume of a cyst (CystV.Max) (mm^3^).

**Fig. 3:**
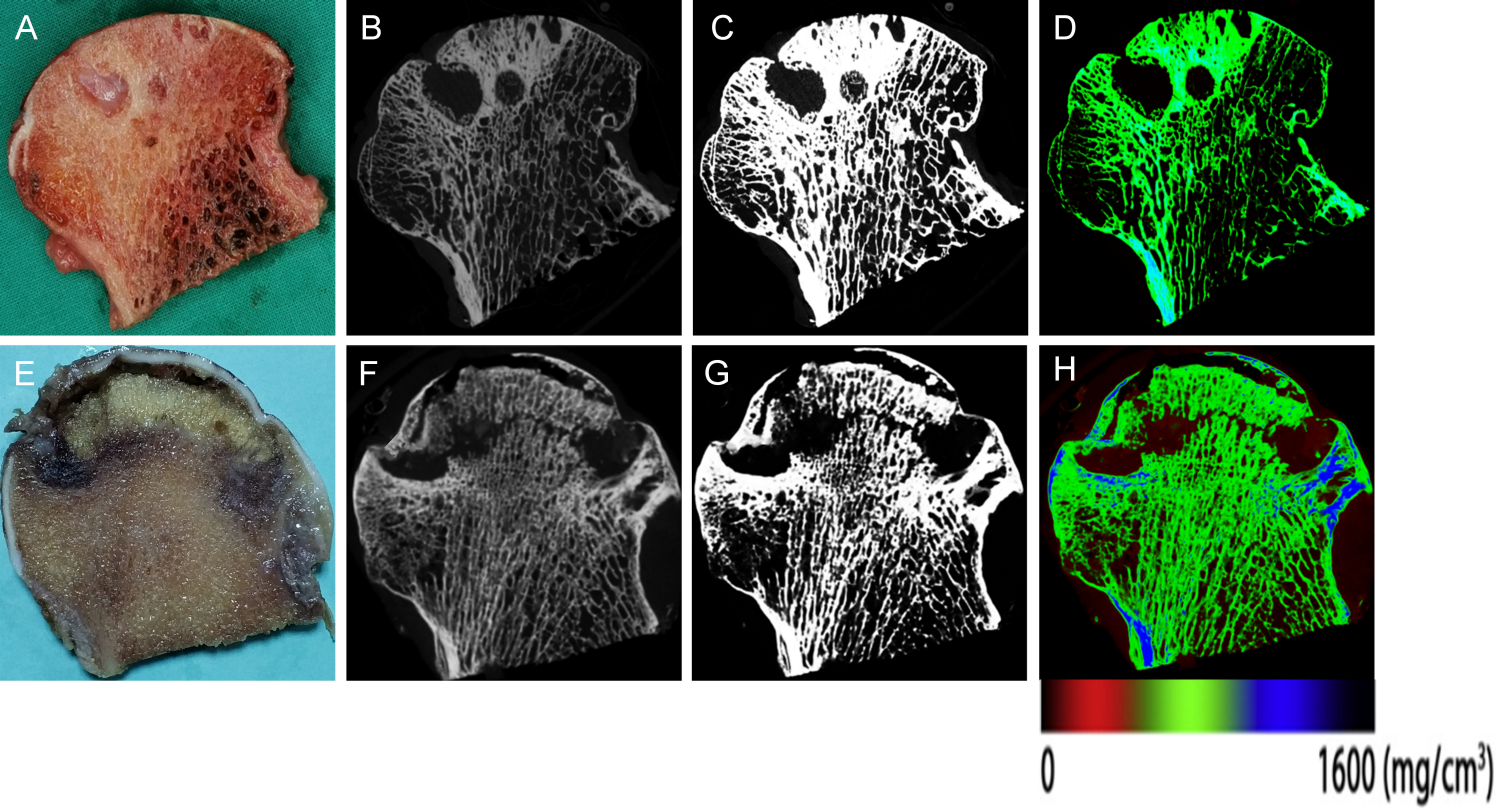
Representative original, binary, and colour micro-CT images corresponding to the coronally sectioned gross specimen of the femoral head. a) to d) Specimens from patients with osteoarthritis. e) to h) Specimens from patients with osteonecrosis of the femoral head. The colour images illustrate mineralization distribution in trabecular bone. Low, intermediate, and high mineral densities are represented by red, green, and blue, respectively.

The distribution of mineralization in the trabeculae was shown by colour images produced using measurements of the X-ray attenuation coefficient (Figure 3). Each measurement region contained regions of interest (ROIs) in the trabecular bone: trabecular bone directly around the bone cyst (Cys-Tb). Cys-Tb is the region extending 0.5 mm from the cyst surface to the trabecular bone.^28^ By using the CTAn software, the following parameters of bone microarchitecture were measured: bone volume fraction (BV/TV) (%), bone surface/volume ratio (BS/BV) (1/mm), trabecular thickness (Tb.Th) (μm), trabecular separation (Tb.Sp) (μm), trabecular number (Tb.N) (1/mm), structure model index (SMI), degree of anisotropy (DA), and connectivity density (Conn.D) (1/mm^3^) (Figure 3).

### Specimen preparation

Following micro-CT examination, all femoral heads were split in half using a diamond saw, and the anterior and posterior hemispheres were coronally separated. The central coronal plane was then cut through each femoral head hemisphere to create a 10 mm-thick bone slice. Decalcification was used to prepare one bone slice for bone histology, while decalcification was not performed on the other slice. All of these specimens were stored in 4% paraformaldehyde for two weeks prior to undergoing the appropriate procedure for undecalcified or decalcified histology. The bone slices were left in the decalcifying solution for a period of several weeks to months, depending on the size and density of the bone tissue. The progress of decalcification was monitored regularly to ensure the complete removal of calcium while preserving the integrity of the bone matrix. This was confirmed by periodic testing, such as the use of a needle to check for residual hardness to ensure the absence of mineral content. Once decalcification was complete, the bone slices were thoroughly rinsed in distilled water to remove any residual decalcifying agent. The specimens were then dehydrated through a series of graded alcohol solutions (e.g. 70%, 80%, 90%, and 100% ethanol) to prepare them for embedding. Decalcified samples were embedded in paraffin wax, cut into 5 μm-thick slices, and stained with haematoxylin and eosin (H&E) and Safranin O. Non-decalcified samples were embedded in methyl methacrylate and cut into 5 μm-thick slices. The sections were then stained using Goldner’s Trichrome method.

### Histopathological assessment and histomorphometry

In this study, histological analysis was specifically focused on the border regions of cystic lesions. This decision was based on the distinct pathological processes associated with ONFH and OA. In ONFH, cystic lesions often result from bone ischaemia leading to necrosis and subsequent collapse of the femoral head. The border regions of these cystic lesions are sites of active bone remodelling and reparative processes, making them critical for understanding the disease mechanisms. In OA, the border regions also exhibit significant bone remodelling activity, which is essential for elucidating the role of cystic lesions in disease progression. Therefore, focusing on the border regions provides valuable insights into the pathological alterations specific to cystic lesions in both conditions.

Histomorphometry was performed on the undecalcified sections using Bioquant Osteo Histomorphometry software (Bioquant Osteo, USA). In order to assess the bone formation and resorption, the following parameters were assessed in each ROI bone: thickness of osteoid (O.Th) (μm), percentage osteoid volume (OV/BV) (%), percentage osteoid surface (OS/BS) (%), specific osteoid surface (OS/BV) (mm^2^/mm^3^), percentage eroded surface (ES/BS) (%), specific eroded surface (ES/BV) (mm^2^/mm^3^), and eroded surface in bone tissue volume (ES/TV) (mm^2^/mm^3^).

On decalcified sections stained with H&E and Safranin O, indications of OA, osteonecrosis, and related pathological changes were evaluated. After immunostaining sections with anti-CD31 (1:100 dilution, AF6191; Affinity, China) antibodies, the degree of angiogenesis was evaluated by light microscopy in accordance with Weidner’s technique.^31^ Microvessels were quantified at 200× magnification. Any brown-stained endothelial cell or cluster of endothelial cells that were clearly isolated from adjacent microvessels, was counted as a single microvessel. Ten representative regions of the section were selected for the count. The examination was conducted by two independent researchers (YM, JY) who were unaware of the group identification.

### SEM analysis

Non-decalcified samples, which were embedded in methylmethacrylate, were sliced into 50 μm-thick slices. Gold-sputtered samples were examined using an SEM (JCM 7000; JEOL, Japan) at acceleration voltages of 15.0 kV to compare the morphological characteristics of cystic lesions in the femoral head between OA and ONFH. The surface of the cystic lesions was scanned and images were captured at different magnifications.

### Statistical analysis

The Statistics Package for Social Sciences was used for the data analysis (SPSS for Windows, version 17.0; SPSS, USA). The Kolmogorov-Smirnov test was performed to determine if the data were normally distributed. An independent-samples *t*-test (parametrical datasets) or the Mann-Whitney U test (nonparametric datasets) was then employed, as appropriate, to determine whether there were any statistically significant differences between OA and ONFH. Continuous data are displayed as mean (SD). The OA and ONFH groups were compared for sex, smoker, diabetes, hypertension, and afflicted side using Fisher’s exact test, chi-squared test, or chi-squared test with Yates correction, which were performed as applicable. A p-value < 0.05 was regarded as statistically significant for all two-tailed hypotheses.

## Results

### 3D distribution of cystic lesions in OA and ONFH

Cystic lesions demonstrated notable distinctions between OA and ONFH. The number of cystic lesions in OA was significantly higher than that in ONFH. CystV.Ave in OA was significantly lower than that in ONFH. In addition, higher values of CystV.Min and CystV.Max in the ONFH were observed. However, no significant difference was found in Cyst.V between OA and ONFH (Supplementary Table i).

In OA, the presence of cystic lesions was significantly more widespread in the femoral head than that in ONFH. While multiple lesions were more prevalent in OA, the difference did not reach significance. Additionally, OA exhibited a notably higher prevalence of femoral heads with more than ten cysts (Supplementary Table i).

According to the three-pillar classification method, zones I and IV were identified as the lateral pillar, zones II and V as the central pillar, and zones III and VI as the medial pillar.^22^ Analysis of cystic lesion locations revealed that in OA, the medial and central pillars exhibited a broader distribution of cysts, while in ONFH, the lateral and central pillars displayed more extensive involvement. It is worth noting that the anterior hemisphere was more affected by cystic lesions, both in OA and ONFH (Supplementary Table ii).

### Comparative analysis of microarchitecture and bone remodelling

**Fig. 4:**
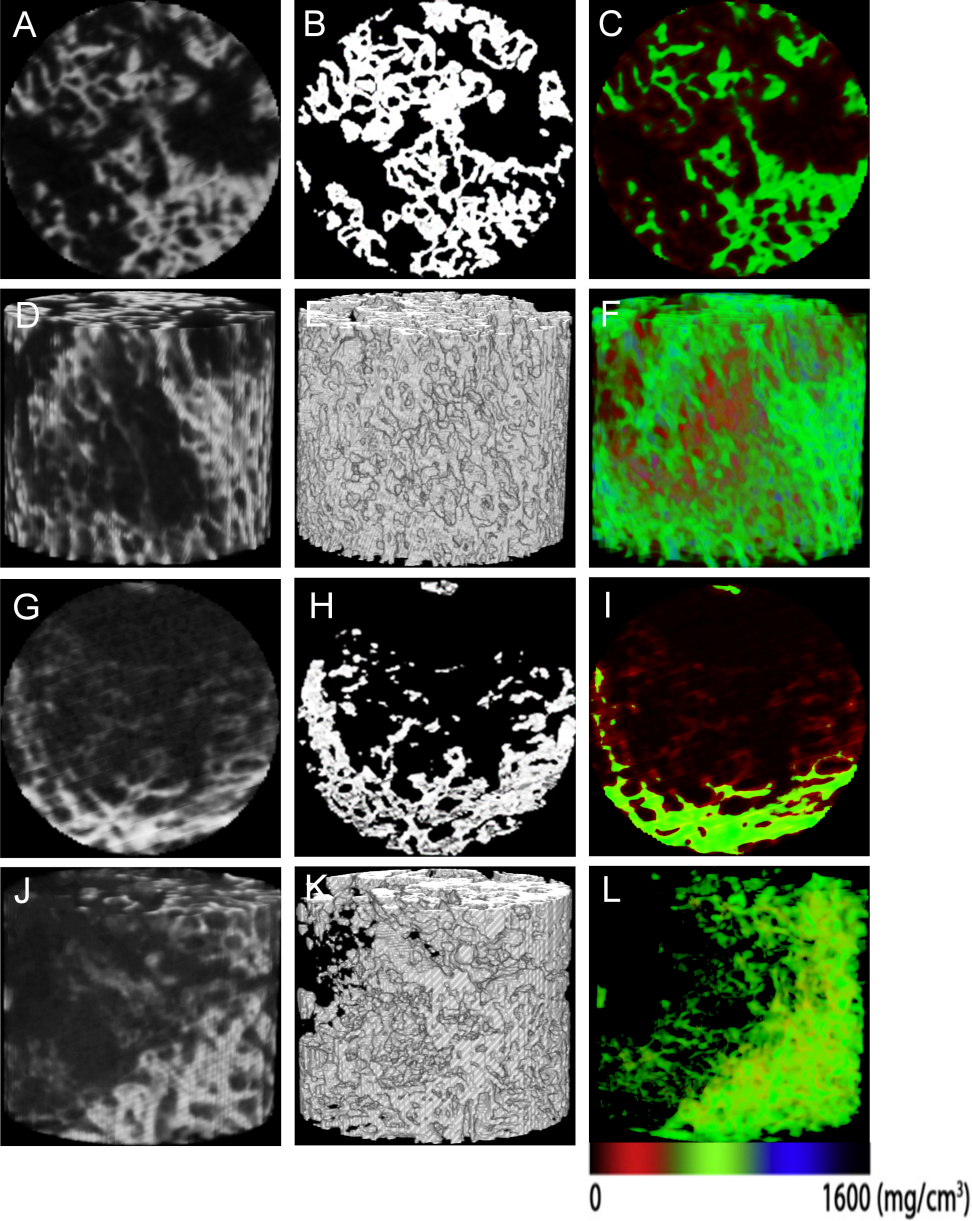
Representative original, binary, and colour micro-CT images of cystic lesions in the femoral head. a) to f) Specimens from patients with osteoarthritis (OA). g) to l) Specimens from patients with osteonecrosis of the femoral head (ONFH). a) to c), g) to i) 2D visualization of the cross-section of the cystic lesions. d) to f), j) to l) 3D reconstruction of the cystic lesions. The colour images illustrate mineralization distribution in trabecular bone. Low, intermediate, and high mineral densities are represented by red, green, and blue, respectively.

In the Cys-Tb region, all the microarchitecture parameters were significantly different between OA and ONFH. Compared to ONFH, there were significantly higher values of BV/TV, Tb.Th, Tb.N, and Conn.D in OA, with significantly lower values of BS/BV, Tb.Sp, SMI, and DA (Figure 4, Supplementary Table iii).

**Fig. 5:**
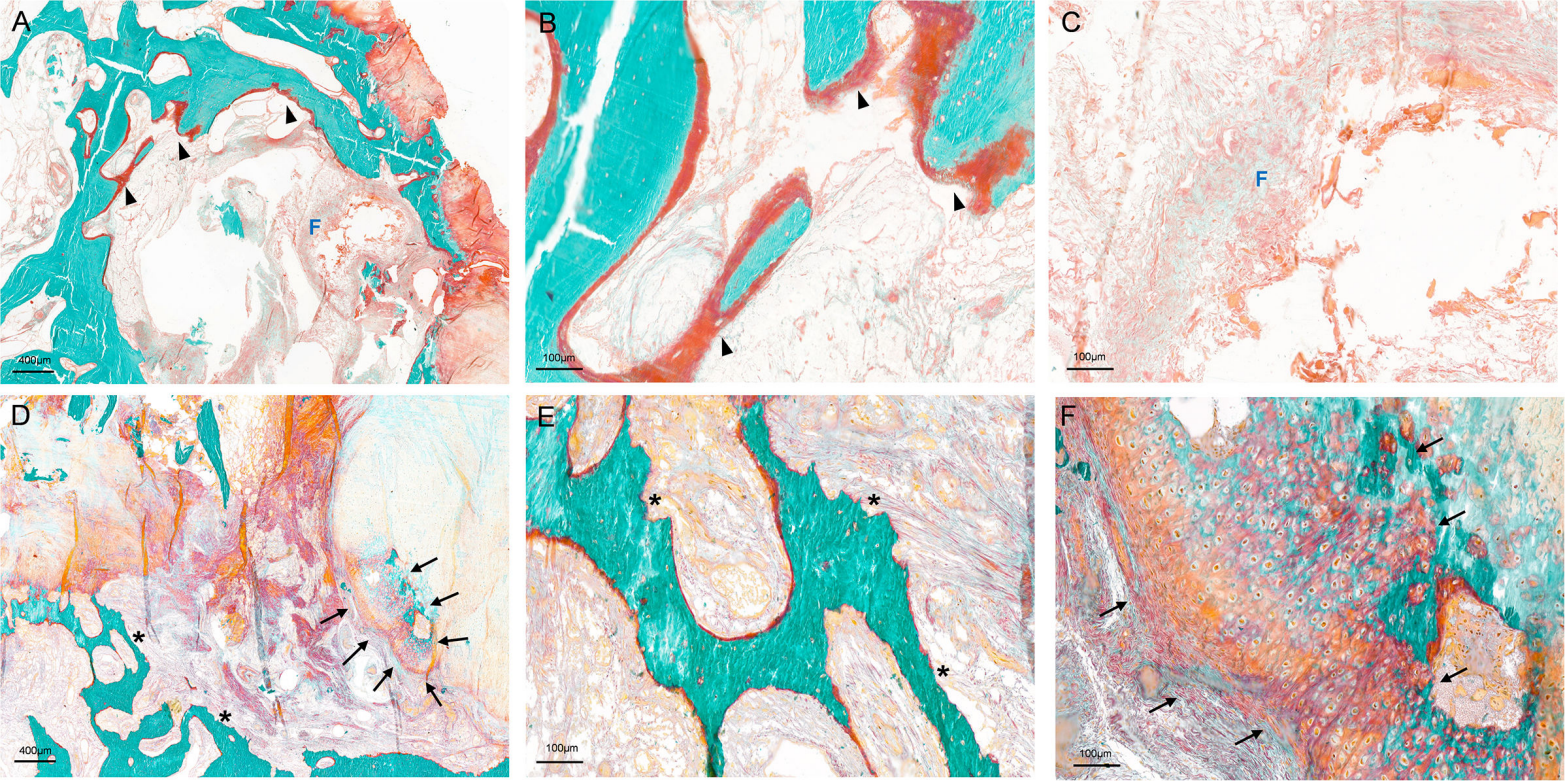
Representative histological findings of cystic lesions in undecalcified sections, focusing on the border regions where active bone remodelling occurs. a) to c) Cystic lesions in the femoral head from patients with osteoarthritis (OA). b) Osteoid (indicated by arrowheads). c) Fibrous tissue (indicated by "F") with collagen texture. d) to f) Cystic lesions in the femoral head from patients with osteonecrosis of the femoral head (ONFH). e) Erosion surface (indicated by asterisk). f) Metaplastic cartilaginous tissue (indicated by arrows). Note: cracks in bone matrix are artifacts resulting from sectioning an uncalcified bone specimen. Stain: Goldner's Trichrome; magnification in a) and d): ×40; magnification in b), c), e), and f): ×200.

In terms of bone remodelling, all the related parameters were significantly different between OA and ONFH (Figure 5, Supplementary Table iii). There were higher values of O.Th, OV/BV, OS/BS, and OS/BV, indicating a more active bone formation status in the Cys-Tb region in OA. However, compared to ONFH, bone resorption activity was much lower in OA, as suggested by lower values of erosion parameters, including ES/BS, ES/BV, and ES/TV.

### Histopathological alterations in cystic lesions

In OA, a bone cyst typically presented as a cavitary lesion with a distinct fibrous lining, encircled by a discontinuous rim of bone structure. These cysts were devoid of an epithelial lining and exhibited homogeneous tissue content. Fibroconnective tissue was the predominant component in the cystic lesions (Figures 5a to 5c).

In ONFH, the reparative process was evident, characterized by the presence of granulation tissue rich in blood vessels. Despite the incomplete reparative substitution of necrotic tissues, angiogenesis was more prevalent in the cystic lesions compared to OA. Numerous dilated veins with thrombi and arteries of various diameters were observed in the periphery areas of the cyst lesion (Supplementary Figure b). Immunostaining with the endothelial cell marker CD31, which is commonly used to indicate angiogenesis,^32^ yielded results consistent with those of H&E staining. Notably, ONFH exhibited a higher presence of arteries with brown-stained cells in the periphery compared to OA (Supplementary Figure b). Additionally, irregular patches containing remnants of metaplastic fibrocartilage were evident (Figures 5d to 5f).

### SEM analysis of cystic lesions

**Fig. 6:**
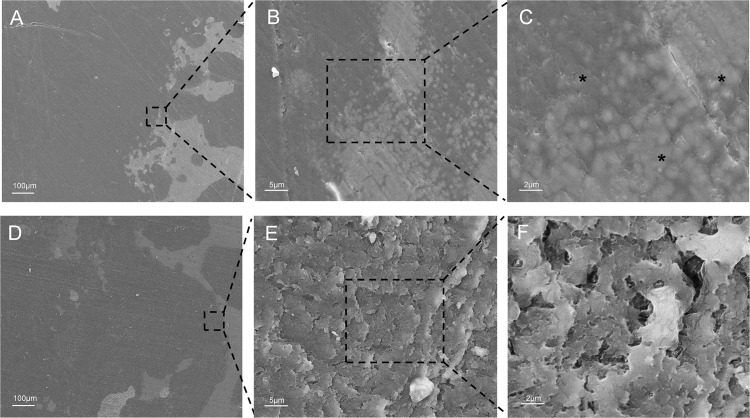
Scanning electron microscopy (SEM) assessment of cystic lesions. a) to c) Representative SEM micrographs of cystic lesions in the femoral head from patients with osteoarthritis (OA). d) to f) Representative SEM micrographs of cystic lesions in the femoral head from patients with osteonecrosis of the femoral head (ONFH). The micrographs were captured at various magnifications (100×, 2,000×, and 5,000×). Multiple spherites of different sizes (indicated by asterisks) with fuzzy peripheries were visible in the OA group.

In the OA group, the spherical particles with fuzzy peripheries were widely dispersed in the surrounding rim of the cystic lesion. Cystic lesions showed a graded distribution of spherical minerals proximal to the periphery trabeculae, culminating in a highly mineralized region (Figures 6a to 6c).

In the ONFH group, cystic lesions were mainly composed of collagenous fibres with different diameters, which were distributed in a random pattern. The collagenous fibres consisted of bundles of twisted collagen fibrils, and were arranged in a mixed weave pattern. At higher magnifications, the shallow concave structures and fissures exhibited irregular shapes. Collagen fibres in the ONFH group appeared to be more contorted than those in the OA group (Figures 6d to 6f).

## Discussion

In this study, we compared the 3D distribution, microstructure, and histopathological features of cystic lesions in OA and ONFH. Our findings revealed significant differences between the two conditions. Cystic lesions in OA were smaller in volume and more densely distributed compared to those in ONFH. While both conditions predominantly exhibited cystic lesions in the anterior hemisphere, ONFH cysts were more prevalent in the lateral pillar, whereas OA cysts were more common in the medial pillar. The trabecular bone surrounding cystic lesions in OA displayed a more sclerotic microarchitecture, characterized by increased bone formation and decreased bone resorption. Histologically, ONFH cysts demonstrated a higher degree of angiogenesis compared to OA cysts. Additionally, SEM analysis revealed a graded arrangement of spherical minerals around OA cysts, while ONFH cysts exhibited more contorted collagen fibres.

The differences in cystic lesion characteristics between OA and ONFH can be attributed to their distinct pathogenic mechanisms. In ONFH, cystic lesions result primarily from bone ischaemia, leading to necrosis and subsequent collapse of the femoral head. The loss of vascular supply triggers a reparative process that often results in angiogenesis, but this process is typically insufficient, leading to continued bone resorption and cyst expansion.^15^ In contrast, OA cysts form due to the infiltration of synovial fluid into the subchondral bone following cartilage injury. Cystic lesions are surrounded by sclerotic bone, which acts as a compensatory response to the degenerative process.^33^ The subchondral bone around the cysts in OA typically undergoes increased bone formation and decreased bone resorption, resulting in a denser, more compact trabecular network.^17^ Both OA and ONFH exhibit a higher prevalence of cystic lesions in the anterior region of the femoral head, likely due to smaller anterior acetabular coverage and the anterior femoral head bearing the majority of force during walking.^34,35^ In ONFH, lesions in the anterior-superior region are a significant risk factor for collapse, particularly when located in the lateral pillar, a critical weightbearing area.^36-38^ The presence of cysts in ONFH often precedes structural collapse, especially when they are large and located in mechanically critical regions.^39,40^ Preservation of the lateral pillar is crucial, as its compromise leads to femoral head deterioration in 95.3% of cases within 0.5 to three years.^41^ Finite element analysis further supports that cystic lesions create localized stress concentration zones, increasing the risk of structural collapse.^42,43^

Histologically, the differences between OA and ONFH further highlight the distinct nature of the cysts and their roles in disease progression. In ONFH, the sclerotic rim around cystic lesions exhibits a weaker microstructure compared to OA, partly due to increased osteoclastic activity in the reactive interface.^44^ This weakening is further exacerbated by the presence of an increased number of blood vessels, which enhance cytokine production, stimulating osteoclast activity and bone resorption.^45,46^ In ONFH, cystic lesions normally consist of loose fibrous materials,^47^ which was consistent with the distorted collagen structure observed by SEM. In contrast, OA cysts are associated with the degradation of mineralization inhibitors (proteoglycans and collagen-II) and increased expression of mineralization promoters (collagen-I, collagen-X, and Runx-2),^48^ leading to calcium phosphate deposition and a graded arrangement of spherical minerals around the cysts.

Cystic lesions in ONFH and OA differ significantly in their clinical implications and management. In ONFH, cysts, especially in the lateral or central pillars, are markers of disease progression and increased risk of femoral head collapse.^22^ Radiological evidence of cysts in these areas necessitates careful monitoring, and early surgical intervention – such as core decompression, bone grafting, or rotational osteotomy – is often required to preserve the femoral head and prevent collapse.^17,49^ Conversely, in OA, cystic lesions form due to cartilage fissures, allowing synovial fluid infiltration into the subchondral bone, primarily in the medial and central pillars, which bear less load.^21,50^ In the early stages of OA, increased stress on the medial cartilage, potentially exacerbated by factors such as injuries to the ligamentum teres, may contribute to cyst development.^51^ These cysts alter subchondral bone mechanics, leading to bone sclerosis and the formation of multiple small cysts.^52^ While OA cysts contribute to joint degeneration, they are not typically associated with acute structural failure. Instead, they are linked to chronic joint pain, stiffness, and reduced mobility.^42,43,53^ As a result, treatment for OA focuses on conservative measures, such as pharmacological therapy and physical rehabilitation,^17^ with THA reserved for advanced stages where the joint function is significantly compromised.^54^ In contrast, the rapid progression and collapse risk in ONFH necessitate more proactive surgical interventions. Our study provides valuable insights into the characteristics of cystic lesions in hip OA, which can contribute to the development of diagnostic and prognostic classifications. The distinct distribution, microarchitecture, and histological features of cystic lesions in hip OA, such as their smaller volume and denser distribution in the medial and central pillars, can serve as potential biomarkers for disease diagnosis. Additionally, the increased bone formation and decreased bone resorption observed in the border regions of cystic lesions may help predict disease progression and guide treatment decisions. Future research could focus on validating these findings in larger cohorts and exploring the integration of advanced imaging techniques to further refine the assessment of cystic lesions in hip OA.

There are some limitations to this study. First, the study was limited by the absence of a control group comprising subjects with normal hip joints or those in the early stage of the disease. Consequently, the findings are reflective solely of the cystic lesion status at the time of surgical intervention. Second, our study did not include tartrate-resistant acid phosphatase (TRAP) staining, which is commonly used to assess osteoclastic activity. Although TRAP staining could have provided additional insights into bone resorption, we chose to use Goldner’s trichrome staining on undecalcified sections in order to simultaneously evaluate both bone formation and resorption. This method allowed us to comprehensively assess the bone remodelling process in the border regions of cystic lesions. Future studies could incorporate TRAP staining to further elucidate the role of osteoclasts in cystic lesion formation and progression. Third, dynamic bone remodelling characteristics were not evaluated in the femoral head specimens in this cross-sectional study. In vivo micro-CT scanning of the hip joint is currently not feasible. Advancements in CT and MRI with enhanced spatial resolution may offer a solution in the future.^55^

In conclusion, our study highlights significant differences in cystic lesion characteristics between OA and ONFH, providing valuable insights into their pathogenesis and clinical management. These findings may enhance the understanding of microstructural, pathological, and histomorphologic alterations of cystic lesions, potentially informing more targeted clinical decision-making for these conditions.

## Data Availability

The datasets generated and/or analyzed during the current study are available from the corresponding author upon reasonable request.
